# Surgical outcome following rotator cuff tear repair in a low-income population. Impact of obesity and smoking

**DOI:** 10.1186/s12891-021-04599-6

**Published:** 2021-08-21

**Authors:** Christine M. M. Silva, Natália M. Mourão, Leila N. da Rocha, Joaquim I. V. D. Landim, Hermano A. L. Rocha, Marco A. A. Lacerda, Francisco A. C. Rocha, José A. D. Leite

**Affiliations:** 1 Postgraduating Program in Surgery, Faculdade de Medicina da Universidade Federal do Ceará, Fortaleza, Brazil; 2 Orthopaedic Service – Shoulder and Elbow Group – Hospital Geral de Fortaleza, Fortaleza, CE Brazil; 3 Department of Internal Medicine, Faculdade de Medicina da Universidade Federal do Ceará, Fortaleza, Brazil; 4 Department of Maternal and Child Health, Faculdade de Medicina, Universidade Federal do Ceará, Fortaleza, Brazil; 5 Department of Global Health and Population, Harvard T. H. Chan School of Public Health, Boston, MA USA; 6 Instituto de Biomedicina – Laboratório de Investigação em Osteoartropatias, Rua Cel. Nunes de Melo, 1315 - 1°. Andar, Rodolfo Teófilo –, Fortaleza, CE 60430-270 Brazil; 7 Department of Surgery, Faculdade de Medicina da Universidade Federal do Ceará, Fortaleza, Brazil

**Keywords:** Shoulder, Rotator cuff tear, Metabolic disease, Obesity, Smoking, Outcome

## Abstract

**Background:**

Comorbidities and socioeconomic issues impact outcome of rotator cuff tear (RCT) repair. There are no data on RCT repair outcome from developing regions. We determined the impact of obesity and smoking following RCT repair in a low-income population.

**Methods:**

This is a retrospective case series. Forty-seven shoulders of 42 patients subjected to open or arthroscopic repair of a RCT with a minimum of 2 years follow-up were cross-sectionally evaluated. Patients were seen in the Orthopaedic Service of the Hospital Geral de Fortaleza-CE, Brazil between March and September 2018. RCT were classified as partial or full-thickness lesions. Fatty infiltration (Goutallier) and tendon retraction (Patte) were recorded as well as obesity (BMI > 30), literacy [>/≤ 8 school years (SY)] and smoking status 6 months prior to surgery (present/absent). Outcomes included pain (visual analogue scale; VAS, 0–10 cm), range of motion [active forward flexion and external rotation (ER)], UCLA and ASES scoring.

**Results:**

Patients were 59.9 ± 7.4 years-old, 35(74.4%) female with 19 (17.1–30.2 IQR) median of months from diagnosis to surgery and 25 median months of follow-up (26.9–34.0 IQR); over 90% declared < 900.00 US$ monthly family income and two-thirds had ≤8 SY. Forty patients (85.1%) had full-thickness tears, 7 (14.9%) had Goutallier ≥3 and over 80% had < Patte III stage. Outcomes were similar regardless of fatty infiltration or tendon retraction staging. There were 17 (36.1%) smokers and 13 (27.6%) obese patients. Outcome was similar when comparing obese vs non-obese patients. Smokers had more pain (*P* = 0.043) and less ER (*P* = 0.029) with a trend towards worse UCLA and ASES scores as compared to non-smokers though differences did not achieve minimal clinically important difference (MCID) proposed for surgical RCT treatment. After adjusting for obesity, VAS and ER values in smokers were no longer significant (*P* = 0.2474 and 0.4872, respectively).

**Conclusions:**

Our data document outcomes following RCT repair in a low-income population. Smoking status but not obesity impacted RCT repair outcome though not reaching MCID for surgical treatment.

## Introduction

Rotator cuff tear (RCT) ranks first among the causes of shoulder pain and dysfunction. Senescence, smoking and history of trauma to the affected shoulder are included as risk factors of having a RCT. In post-mortem studies, the prevalence of partial and/or full-thickness RCT has been shown to vary from 5 to 40% and imaging studies found that individuals younger than 40 rarely display a RCT while up to 50% in their 80s may present those lesions [1].

Patients with acute posttraumatic RCT lesions are the best candidates for surgical repair, but those younger than 65 years of age presenting with symptomatic larger lesions (> 1–1.5 cm) are highly considered for surgery [1, 2]. Although comorbidities were shown to modify the clinical picture of patients with a RCT, few studies addressed their impact on the outcome after surgical repair [3]. Being a current heavy smoker, meaning over 20 pack-year, was associated with increased prevalence and severity of RCT [4]. A retrospective study evaluating the influence of cigarette smoking in RCT found a positive association with increased severity at presentation and an inferior response to treatment, as compared to non-smokers [5]. More recently, smoking was shown to be associated with healing failure following RCT surgical repair [6]. It has also been proposed that obesity affects both prevalence and severity of RCT lesions. However, concerning surgical results, there is controversy as to whether obesity impact RCT repair outcomes [7]. Socioeconomic issues, including work compensation, were considered relevant both regarding disease presentation as well as response to treatment in patients with RCT [8]. However, there is a paucity of data on the treatment of RCT in developing regions. Our orthopaedic service deals with a low-income population that has difficult access to tertiary services and diagnosis. As a result, time from diagnosis to surgery can be delayed and access to rehabilitation facilities is far from ideal [9]. Given that those shortcomings could affect surgical results in patients subjected to RCT repair we studied the outcome following at least 2 years of RCT repair in a low-income, low-literacy population.

## Methods

Clinical and demographic data from 42 patients with a diagnosis of RCT that were subjected to open or arthroscopic surgical repair following at least 2 years follow-up were cross-sectionally evaluated. A total of 47 surgeries were included since 5 patients had both shoulders operated at different time points. Patients were consecutively seen from March 2018 to September 2018 at the Shoulder and Elbow Division of the Orthopaedic Service of the Hospital Geral de Fortaleza, CE, Brazil. The clinical protocol was approved by the local Ethics Committee (protocol number: CAEE 97223018.0.0000.5040) and all patients signed an informed consent. All procedures were performed according to relevant guidelines. Patients were directly interviewed and subjected to clinical examination and hospital files were revised. A diagnosis of a partial or full-thickness RCT was established using Magnetic Resonance Imaging, which was confirmed by intraoperative evaluation. Patients with a prior history of surgery in the index shoulder, fracture in the ipsilateral superior limb or infection were excluded. Surgeries were performed by two board-certified experienced surgeons (CMMS, MAAL). Although results can differ depending on the operation methods and/or surgeon, this bias is probably minimized in our setting as only these surgeons, which work together and follow standardized protocols, performed the procedures. Briefly, patients were operated in a lateral decubitus position under general anesthesia and brachial plexus blockade. A single or a double-row repair was chosen during surgery depending on the RCT lesion*.* Those in which a biceps lesion was identified during surgery were also subjected to tenotomy or tenodesis. The operated arm was kept in an anti-rotation sling. Passive range of motion was done starting in the first day following surgery. After 6 weeks, active physiotherapy was initiated. Patients were instructed before hospital discharge on how to perform exercises but adhesion to this practice was not recorded. Shoulder lesions were characterized regarding fatty infiltration using Goutallier staging and degree of tendon retraction using the Patte classification, as described previously [10]. Patients were asked about monthly family income and data are presented using March 2020 as reference for conversion of Brazilian to US$ currency. Working activities were classified concerning predominant white or blue-collar jobs. Patients declaring no practice of regular exercise were considered sedentary. Socioeconomic data did also include literacy, registering patients-declared years of school education [>/≤ 8 school-years (SY)]. Body mass index (BMI) was calculated as weight / squared height (kg / m^2^) and obesity was defined as BMI > 30. Smoking status was evaluated whether present or absent considering the previous 6 months prior to surgery. Pain intensity was calculated using a visual analogue score (VAS, 1–10 cm). Outcomes did also include the University of California at Los Angeles score (UCLA; 35 = best score) and the American Shoulder and Elbow Surgeons score (ASES; 100 = best score) which have been validated to Portuguese [10–12]. Shoulder range of motion considered degrees of maximal active forward flexion (FF) and external rotation (ER).

### Statistical analysis

Data are presented as means (Standard deviation, SD) or medians (Interquartile range, IQR) and comparison between groups was done using Student’s t-test and Kruskal-Wallis for comparison of means or medians, as appropriate. Assessment of normality of continuous data was done using the Kolmogorov-Smirnov test. No imputation was done for missing data. A multivariate analysis of smoking, adjusted for obesity, was performed regarding specifically VAS score and maximal ER, using generalized linear models with robust error estimators only with main effects terms in the model. The level of significance was set at 0.05. Entering of data and univariate data analysis were done using SPSS v17, SPSS Inc.

## Results

Table 1 displays demographic and clinical data of 47 RCT surgical repairs performed in 42 individuals. Almost two-thirds of the procedures were arthroscopic. Mean age at the time of surgery was 59.9 ± 7.4 years (range, 51–83 years) being 35 (74.4%) female. Median time from diagnosis to surgery was 19 months (IQR 17.1–30.2). Patients had a median of 25 months follow-up (26.9–34.0 IQR). The low-income characteristic is illustrated by the fact that over 90% of patients declared family monthly earnings below 900.00 US$. Additionally, roughly two-thirds of our patients declared less than 8 years literacy (Table 1). The dominant shoulder was involved in 34 (72.3%) of the patients. Obesity was present in roughly one-quarter of the patients and 17 (36.1%) were smokers. Most patients [39 (82.9%)] denied practice of regular physical activity. Cardiovascular comorbidities and osteoarthritis were very prevalent (Table 1).

**Table 1:** Clinical characteristics of 47 low-income patients subjected to Rotator Cuff Tear (RCT) surgical repair

Characteristic	N(%)
Female	35 (74.4%)
Age (mean ± SD)	59.8 ± 2.38
RCT Cause	
Degenerative RCT Lesion	29 (61.7%)
Trauma	18 (38.2%)
Full-thickness tear	40 (85.1%)
Dominant side operated	34 (72.3%)
Sedentary	39 (67%)
Arthroscopic repair	31 (65.9%)
Smokers	17 (36.1%)
Literacy	
≤ 8 years	32 (68%)
> 8 years	15 (31.9%)
Monthly family income	
US$ < 300	27 (57.4%)
300 < US$ < 900	17 (36.1%)
US$ > 900	3 (6.3%)
Occupation	
Blue Collar	42 (89.3)
White Collar	05 (10.6)
BMI	
Normal	9 (19.1%)
Overweight	25 (53.1%)
Obese	13 (27.6%)
Comorbidities	
Cardiovascular disease	03 (6.3%)
Diabetes mellitus	12 (25.5)
Dyslipidemia	20 (42.5%)
Hypertension	21 (44.6%)
Hand ao	12 (25.5%)
Knee ao	17 (36.1%)
Previous shoulder corticosteroid infiltration	14 (29.7%)

Data represent mean (SD) and N(%) of 47 low-income patients subjected to RCT repair.

*BMI* body mass index, *OA* osteoarthritis, *RCT* rotator cuff tear

Most RCT lesions were deemed degenerative and the vast majority [40 (85.1%)] had full-thickness tears. Mean (SD) and median time from trauma to surgery was 13.7 ± 5.6 and 9 (IQR 5–16) months, respectively. Thirty patients had an isolated supraspinatus lesion, 7 had both supra and infraspinatus lesions, 6 had both supra and subscapular lesions and 4 displayed combined supraspinatus, infraspinatus and subscapular lesions. Although most [40 (85.1%)] had mild-moderate fatty infiltration (Goutallier stage 0–2) there were 7 (14.9%) patients with prominent fatty streaks (Goutallier stage 3). Retraction was also mild-moderate in most patients but 9 (19.1%) had a severe grade III retraction. There was no association of either fatty infiltration or tendon retraction with surgical outcomes (Table 2).

**Table 2:** Impact of fatty infiltration and tendon retraction in RCT repair

	UCLA	VAS	ASES
Patte 1	25.6 (6.1)	5.5 (3.1)	45.5 (31.8)
Patte 2	26.3 (7.3)	3.8 (2.8)	56.4 (32.3)
Patte 3	27.7 (7.2)	3.2 (3.3)	60.6 (38.6)
*P* value	0.702	0.217	0.331
Goutallier 0–2	25.2 (7.1)	4.5 (3.0)	49.3 (32.7)
Goutallier 3–4	28.5 (9.5)	2.4 (3.0)	70 (37.8)
*P* value	0.172	0.086	0.109

47 low-income patients were subjected to rotator cuff tear (RCT) surgical repair. Data represent mean (SD), *ASES* American Shoulder and Elbow Shoulder Score, *BMI* body mass index, *ER* external rotation, *FF* forward flexion, *UCLA* University of California at Los Angeles shoulder score, *VAS* visual analogic scale.

Patients classified as obese had similar outcomes regarding pain values, UCLA and ASES scores when compared to non-obese individuals. Neither were there any differences in measurements of shoulder range of motion when comparing obese vs non-obese patients (Table 3).

**Table 3:** Impact of Obesity in RCT repair in low-income patients

	BMI ≥ 30 kg/m^2^	BMI < 30 kg/m^2^	Mean difference (95% CI)	*P* value
UCLA	25.3 (7.8)	27.2 (6.6)	1.9 (−2.6, 6.4)	0.076
VAS	4.8 (2.7)	4.0 (3.3)	0.8 (−1.2, 2.8)	0.196
ASES	57.5 (28.8)	62.2 (30.9)	4.7 (−15.2, 24.6)	0.322
Active FF (°)	133.1	137.3	0.61 (−25.4, 26.6)	0.365
Active ER (°)	43.0	48.3	5.31 (−6.5, 17.1)	0.124

47 low-income patients (13 with BMI ≥ 30 kg/m^2^; 34 with BMI < 30 kg/m^2^) were subjected to rotator cuff tear (RCT) surgical repair. Data represent mean [SD], *ASES* American Shoulder and Elbow Shoulder Score, *BMI* body mass index, *ER* external rotation, *FF* forward flexion, *UCLA* University of California at Los Angeles shoulder score, *VAS* visual analogic scale.

On the other hand, pain values had mild though significantly increased values in smoking vs non-smoking patients (Table 4). Regarding range of motion, FF values did not differ but ER values were significantly higher in non-smokers as compared to smokers. Outcome results in smokers did also show a trend towards worse UCLA and ASES scores as compared to non-smokers, although not reaching statistical significance.

**Table 4:** Impact of Smoking in RCT repair in low-income patients

	Smoking	Non- Smoking	Mean difference (95% CI)	*P* value
UCLA	24.2 (5.9)	27.3 (7.5)	3.1 (−1.1, 7.3)	0.059
VAS	5.3 (2.8)	3.7 (3.2)	1.6 (− 0.2, 3.4)	0.043
ASES	52.9 (27.6)	65.6 (31.0)	12.7 (−5.5, 30.9)	0.085
Active FF (°)	135.8° (36.7)	139.8° (41.1)	4 (−20.2, 28.2)	0.372
Active ER (°)	40.2° (20.4)	50.6° (13.3)	10.4 (0.5, 20.2)	0.020

47 low-income patients were subjected to rotator cuff tear (RCT) surgical repair. Data represent mean (SD), *ASES* American Shoulder and Elbow Shoulder Score, *BMI* body mass index, *ER* external rotation, *FF* forward flexion, *UCLA* University of California at Los Angeles shoulder score, *VAS* visual analogic scale.

After performing a multivariate analysis (generalized linear models with robust error estimators) of smoking adjusted for obesity, there was no statistically significant difference for VAS score and Active ER, with *P* = 0.055 and 0.061, respectively.

We also performed an analysis considering the number of tendons involved, specifying isolated supraspinatus, double lesion (supraspinatus plus infraspinatus or supraspinatus plus subscapularis) or triple lesion, which returned no significant difference in surgical outcome, as shown in Table 5.

**Table 5:** Outcome of RCT repair according to the number of injured tendons

	SS	DB	TT	*P* value
UCLA	25 (1.5)	26.7 (2.3)	28.5 (1.8)	0.4816
VAS	4.3 (0.6)	4.0 (1.0)	3.6 (1.3)	0.8613
ASES	57.7 (5.7)	65.8 (11.4)	59.6 (11.1)	0.7927

47 low-income patients were subjected to rotator cuff tear (RCT) surgical repair and classified as having isolated supraspinatus (SS; *n* = 29), double (DB; *n* = 10) or triple (TT; *n* = 8) tendon lesions (see text for details). Data represent mean (SD), ASES American Shoulder and Elbow Shoulder Score, *UCLA* University of California at Los Angeles shoulder score, *VAS* visual analogic scale; *P* value evaluated using one-way ANOVA followed by Tukey test, if appropriate.

Surgical outcome was similar with both surgeons. Mean (SD) UCLA/ASES scores were 27.7(1.3)/63.5(6.4) and 24.4(1.9)/58.3(6.6) in 25 and 22 patients operated having MAAL or CMMS as the main surgeon (*P* = 0.1633 and 0.5726 for UCLA and ASES values between surgeons), respectively. Mean (SD) pain levels also did not differ, being 3.7(0.6) and 4.7(0.7) in patients operated by MAAL and CMMS, respectively (*P* = 0.3597). There were no relevant postsurgical complications including no infections. One patient had a shoulder luxation of an operated limb following trauma.

## Discussion

This is the first study reporting surgical outcomes in a low-income population subjected to RCT repair. Our data reveal that patients classified as obese had a similar outcome as compared to non-obese patients whereas those that were current smokers apparently behaved worse. The majority of the individuals in our cohort belonged to a very low-income population based on the declared family income. Additionally, the fact that most had blue-collar occupations and displayed a very low-literacy corroborate the low-income profile.

Obesity has been associated with healing issues and increased post-surgical infection rates [6]. Additionally, being overweight has been both associated with increased prevalence and severity of RCT and need for surgical repair [13, 14]. However, in addition to the low number of reports addressing the impact of obesity on outcomes following RCT repair, the few existing publications gave conflicting results [7]. Two retrospective studies with a relatively short-term follow-up found that obesity either had no impact on RCT surgical results or was significantly associated with increased hospital stay and operative time as well as worse functional outcomes [15, 16]. Another retrospective study evaluating prospectively collected data of a registry found no difference regarding surgical outcome when comparing obese with non-obese individuals [7]. Due to mechanical issues, obesity has been regarded as a prominent damage factor to weight bearing joints, which could possibly be less relevant for the shoulder even if it can be argued that simply getting out of a chair or bed could represent overload to the shoulder of an obese patient. Indeed, although persistent mechanical damage has been implicated in the development of damage to shoulder tendons, such as it happens in some professional or sport activities, being spared from weight impact would at least partially protect that joint from mechanical damage [7]. However, a systemic inflammatory component secondary to the release of inflammatory mediators produced by adipocytes, namely adipokines and inflammatory cytokines such as interleukin (IL)-6, has also been implicated in the pathogenesis of musculoskeletal disease. In keeping with this assumption, diabetes, dyslipidemia and metabolic syndrome, which have been listed among comorbidities impacting the natural history of RCT lesions and are associated with obesity, have their pathogenesis linked to increased release of those inflammatory mediators [3, 17]. There are assumptions that severely obese patients display less shoulder range of motion that could spare the joint thus favoring patient reported outcomes. We were not able to address this issue given that we had no such patients included in this study. Actually, given that our patients had predominantly blue-collar jobs we believe they had a higher need for mechanical use of the joint. Be as it may, we were relieved by the fact that our patients restored their daily living activities following surgical recovery (data not shown).

Smoking has been associated with worse clinical scores in patients with a RCT. Various studies have associated smoking with adverse results following surgical orthopaedic procedures lending to the idea that it should also impact RCT repair [5]. That is not say that surgery is contra-indicated in smoking patients but they may achieve lower results. Using retrospective data, it was shown that smokers had a significant improvement following RCT repair, though milder than results achieved by non-smokers [18]. Our data revealed that smokers had a trend toward worse outcome following RCT repair. Although only mean pain values and degree of external rotation reached statistical significant difference when comparing results in smokers vs non-smokers, both UCLA and ASES scoring showed a trend toward difference favoring the non-smoker group. However, when adjusted for obesity, smoking habit was no longer associated with worse results following RCT repair. Similar to previous studies, we were unable to separate groups regarding quantitation of the smoking habit because we would need a larger sample size [5]. Therefore, our analysis was limited as a dichotomous variable censoring 6 months prior to surgery. Multiple factors are probably at stake when attempting to address the relevance of smoking to a surgical result. Not only would smoking promote alterations in joint tissues but also affect healing following surgery [19, 20]. It is probably beyond doubt that smoking cessation would be of help prior to surgery [5]. However, the fact that smokers apparently achieve less benefit following RCT repair should not preclude indication of an operative treatment. A shared decision between patient and health care professionals involved is probably the best alternative to improve results and patient satisfaction.

Statistical significance does not necessarily mean clinical relevance. Patient reported outcomes have been increasingly considered when evaluating a therapeutic decision leading to the development of minimal clinically important differences (MCID) to analyze outcomes. There are two studies defining MCID for conservative or surgical therapy of patients with a RCT. Higher values, defined as 27.1 and 2.4 for ASES and VAS scores, meaning greater improvement were required to meet a MCID in patients subjected to an operative procedure, which could be partially due to greater expectations in patients willing to undergo surgery [21, 22]. Using the proposed criteria for surgical RCT treatment, the outcome difference observed in our study between smoking and non-smoking patients, which were 12.7 and 1.6 for the ASES and VAS scores, respectively, did not achieve MCID [22].

Health inequities refer to differences among individuals related to socioeconomic issues, usually representing an additional burden to those living in unfavorable conditions since their work force is heavily compromised when sick [9, 23]. A very recent population-base cohort study found that increased age, male gender, obesity and social inequities represented by a lower deprivation score and having blue-collar occupation was associated with an increased need for RCT surgery [14]. However, we are not aware of previous studies focusing on inequities and its impact in surgical outcome following RCT repair. Our study was performed in a very low-income region, with a mean GDP/capita below US$ 4500.00 which is mirrored by the fact that almost 90% had blue-collar jobs and, unfortunately, a low-literacy profile. Monthly family income data considered values for less than 1, between 1 and 3, and over 3 minimum salaries. However, there are large inequities regarding income in Brazil. Using official data from 2018, the mean per capita income in Fortaleza, located in the northeast region, was US$ 6939 as compared to US$ 16,061 in São Paulo, located in the wealthier southeast region of the country [24]. Though there are no such data from other cohorts, our mean income numbers are likely much lower than mean GDP/capita from wealthy regions [9]. All patients were treated in the public service, which covers over 85% of the Brazilian population, with no direct payments. We believe that difficult access to a tertiary center, delay in time to surgical intervention and limitations in access to rehabilitation facilities are more prominent among our patients [9, 25]. Social inequities probably affected outcomes in our study. The mean ASES and pain scores in our cohort were 59.85 and 4.4, with a mean of 100 weeks follow-up, as compared to 71.3 in a study conducted in Michigan with over 3 years follow-up [7]. Also, a study from Utah reported 80.8 and 1.8 mean for ASES and pain scores, respectively, after 1 year (52 weeks) follow-up [22] and outcome also in a high-income cohort from Norway revealed a mean 85.4 ASES score in patients subjected to surgery after failing physiotherapy with 5 years follow-up [2]. Median annual per capita income are reported to be US$31,771 and US$32,892 in Utah and Michigan, USA, respectively [26], as compared to values below US$4500 in our cohort. We also speculate that differences in follow-up time, patient selection, presence of comorbidities and type of lesions may account for discrepancies between these results*.*

Limitations of this study include the low number of patients. Also, data were collected cross-sectionally, which limits appreciating causality. However, in addition to the low number of studies evaluating the effect of comorbidities on the outcome of RCT repair, we believe there are none from developing regions. We also did not assess healing rate, since imaging examinations were requested only in patients with persistent pain following RCT repair. We also could not quantitate smoking habit because of the low number of patients. This might be relevant as smoking prevalence is lower in our country as compared to data from Europe and we saw a steady smoking habit decline in Brazil in the last 10 years, in both genders [27]. Smokers were shown to present larger RCT lesions and being younger at presentation in addition to achieving less benefit following surgery [5]. The fact that the difference between our smoking and non-smoking groups did not achieve MCID may be due to our low number of patients as well as to the fact that we did not quantitate smoking. Although we may argue that smoking cessation is a healthy initiative, there is a need for more data to determine smoking impact on RCT surgery.

## Conclusion

Low-income patients subjected to RCT repair achieve significant improvement with an acceptable safety profile. Obesity does not appear to impact surgical outcome but being a current smoker appears to be associated with achievement of inferior results. The degree of fatty infiltration and tendon retraction were not associated with outcome following surgical RCT repair in this cohort.

## Data Availability

All data generated or analysed during this study are included in this published article.

## Abbreviations

ASES: American Shoulder and Elbow Surgeons score
BMI: Body mass index
CI: Confidence interval
ER: External rotation
FF: Forward flexion
IQR: Interquartile range
MCID: Minimal clinically important differences
RCT: Rotator cuff tear
SY: School-years,
SD: Standard deviation
UCLA: University of California at Los Angeles score
VAS: Visual Analogue Scale
